# *Pfhrp2* Deletions in the Democratic Republic of Congo: Evidence of Absence, or Absence of Evidence?

**DOI:** 10.1093/infdis/jix345

**Published:** 2017-07-22

**Authors:** Charles J Woodrow, Caterina Fanello

**Affiliations:** 1 Mahidol Oxford Research Unit, Faculty of Tropical Medicine, Mahidol University, Bangkok, Thailand; and; 2 Centre for Tropical Medicine and Global Health, Nuffield Department of Medicine, University of Oxford, United Kingdom

To the Editor—Parr et al report that *Plasmodium falciparum* parasites with deletion of the *pfhrp2* gene can be found in children across all provinces of the Democratic Republic of Congo [1]. It is possible to differentiate gene deletion from other causes of a negative *P. falciparum* histidine-rich protein 2 (PfHRP2)–based rapid diagnostic test via a series of investigations [2, 3], starting with positive microscopic identification of *P. falciparum*, a straightforward way of minimizing at the outset the chance that the gene cannot be detected simply because of low levels of DNA [4]. Parr et al used many of these recommended steps, and the work was clearly undertaken to a high technical standard, but a distinct feature of the study was that the diagnosis of *P. falciparum* was determined by real-time polymerase chain reaction (PCR) detection of *pfldh*, a relatively sensitive approach designed to capture all *pfhrp2*-deleted parasites. The article comes to a confident conclusion that the *pfhrp2*-negative PCR results represent gene deletion rather than insufficient DNA.

Was this confidence justified? The answer, in our view, is a clear “no.” Unsurprisingly, a large proportion (90%) of the “*pfhrp2*-deleted” samples were negative on microscopy. In a set of samples where parasitemia is generally below the level of microscopic detection, successful PCR amplification of a control gene does not guarantee that another gene will be robustly amplified from the same sample. Borderline DNA concentrations will cause stochastic failure of individual PCR reactions, so samples negative by PCR for *pfhrp2* but positive for other genes and, hence, fulfilling the study’s criteria for “*pfhrp2* deletion,” will be inevitable simply because of the number of samples studied.

The potential for overcalling “deletions” gets even worse if any of the positive control PCR reactions are more sensitive than the test reactions. No data on relative sensitivity of the *pfhrp2* and control gene reactions were provided (in fact, optimal conditions were explored during the course of the study). However, there is evidence for differential sensitivity of the 2 *pfhrp2* PCR reactions. Of the 91 samples positive for only 1 of the 2 *pfhrp2* sequences (calculated from supplementary data), most (80) were positive at exon 1/2, with 11 positive at exon 2. The obvious explanation for this, with DNA limiting, is that the exon 1/2 PCR had greater sensitivity: it involved nested primers and a 308-bp amplicon whereas the PCR for exon 2 involved a single pair of primers and a longer amplicon (Table 1), both factors associated with reduced PCR efficiency [5]. Instead, it is simply assumed that all these isolates represent partial *pfhrp2* deletions, despite the absence of reports of such parasites in large surveys [6, 7].

**Table 1.** Characteristics of Polymerase Chain Reaction Assays Used in the Study of Parr et al

Sequence, Section	Length in Genome (Outer Primers)	Polymerase Chain Reaction Type
*pfhrp2*, exon 1/2	308	Nested
*pfhrp2*, exon 2	842	Single pair of primers
*pfhrp3*, exon 1/2	301	Single pair of primers
*pfhrp3*, exon 2	719	Heminested
β-tubulin	77	Single pair of primers
*pfldh* ^a^	Not applicable	Real-time

^a^Positive result used as entry criterion to study.

In addition, there was an intrinsic bias in study design. For a sample to avoid the classification “*pfhrp2* deleted,” both *pfhrp2* sections had to be amplified. But subsequent “confirmation” of sufficient DNA required that only 1 of 3 control PCR sequences be amplified (2 sections of *pfhrp3* and β-tubulin). The authors state that of “149 *pfhrp2*-deleted *P. falciparum* isolates, only 5 (3.4%) had co-existing complete *pfhrp3* deletions,” but this is the wrong comparison—to interpret the data properly, we need to know how many samples had negative results for *either* of the *pfhrp3* sections (the standard applied for *pfhrp2*). Although this was not directly determined (control PCR assays were undertaken serially until a positive was obtained), the supplementary data show that the first *pfhrp3* PCR failed in 73 cases, again indicating broadly low levels of DNA.

The finding that a subset of samples with “*pfhrp2* deletion” had a higher proportion of PCR failures at 2 neighboring microsatellite markers is offered as further evidence for gene deletion. Again, the evidence that this was not simply due to low DNA concentrations is not compelling. Markers were approximately evenly amplified in control samples, but these are likely to have had substantially higher levels of DNA. The lower DNA levels that were certainly present in the *pfhrp2*-negative samples could have exposed lower PCR efficiency at 2 particular markers. Notably, these markers are on the opposite side of the *pfhrp2* gene compared with markers absent in *pfhrp2*-deleted parasites from Latin America [8].

In summary, the concern that many, and possibly all, of the “deletion” samples are just samples with low levels of DNA is never satisfactorily dispelled. This is a much more plausible explanation for their ubiquitous distribution across the country. For these reasons, calls for alternatives to PfHRP2 as a diagnostic antigen in this region are not yet indicated.

## Note

***Potential conflicts of interest.*** Both authors: No reported conflicts of interest. Both authors have submitted the ICMJE Form for Disclosure of Potential Conflicts of Interest. Conflicts that the editors consider relevant to the content of the manuscript have been disclosed.
